# Isolated hypoglossia: Oromandibular Limb Hypogenesis Syndrome Type 1 A – A Rare Case Report

**DOI:** 10.4317/jced.62077

**Published:** 2024-12-01

**Authors:** Manish Jha, Pooja Singh, Anusree Paul, Gopal Chandra Bera

**Affiliations:** 1 Associate Professor, Department of Orthodontics & Dentofacial Orthopaedics, GNIDSR, Kolkata, India; 2 Associate Professor, Department of Pediatric & Preventive Dentistry, K.S.D. Jain Dental College & Hospital, Kolkata, India

## Abstract

Hypoglossia is a rare developmental anomaly of tongue. It is usually associated with various syndromes and other anomalies. Most common association of hypoglossia is with limb deformity and these disorders are collectively grouped as Oro Mandibular Limb Hypogenesis (OLHS) Syndrome. It represents a spectrum of disorders and cases with deviation from the original classification has been reported. Isolated hypoglossia without limb deformity is very rare and has been classified as OLHS Type 1 A by Hall. Features associated with this disorder is hypoplastic mandible, absence of mandibular incisors, intra oral bands and marked enlargement of sublingual muscular ridges and glands. This article reports a case of 22 years old female patient with isolated hypoglossia. Patient reported with the chief complain of irregularly placed teeth. Extra oral finding revealed a convex profile with retrognathic mandible. Intraoral examination revealed crowding in teeth with a constricted maxillary and mandibular arch. A small rudimentary tongue with reduced range of movement was seen. However no abnormalities of the extremities was seen.

** Key words:**Hanhart syndrome, Hypoplastic mandible, Oligodontia, Small tongue.

## Introduction

Anomalies of tongue can be divided in two broad categories – the acquired and developmental types. Common acquired anomalies are inflammatory disorder like oral lichen planus and glossitis of tongue, infectious lesions like candidiasis, leukoplakia, autoimmune disease like Sjogren syndrome. Common developmental anomalies include aglossia, microglossia, macroglossia, accessory tongue, bifid tongue, glossitis rhombica mediana and lingual thyroid nodule (1). Aglossia means absence of tongue however in hypoglossia/ microglossia a small rudimentary tongue is present. Both aglossia and hypoglossia are rare congenital anomalies and are usually associated with limb abnormalities or are a part of various syndromes like Moebius syndrome, Hanhart syndrome, Charlie M syndrome, Hypoglossia Hypodactylia syndrome (2). Hypoglossia was first reported by de Jussieu in 1719 after which around few cases with and without limb abnormalities have been reported (3) but isolated cases of hypoglossia though reported (4,5) are very rare. In the last two centuries less than 30 cases of isolated hypoglossia have been reported (6).

The case presented here is of isolated hypoglossia without any limb abnormalities.

## Case Report

**Figure 1:**
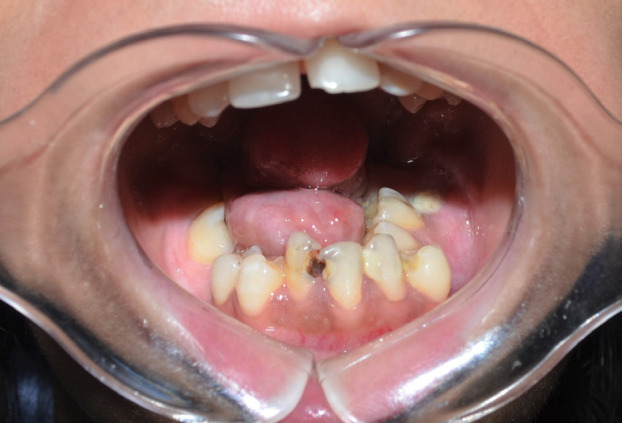
Intra oral picture showing constricted mandibular arch and a small rudimentary tongye.

**Figure 2:**
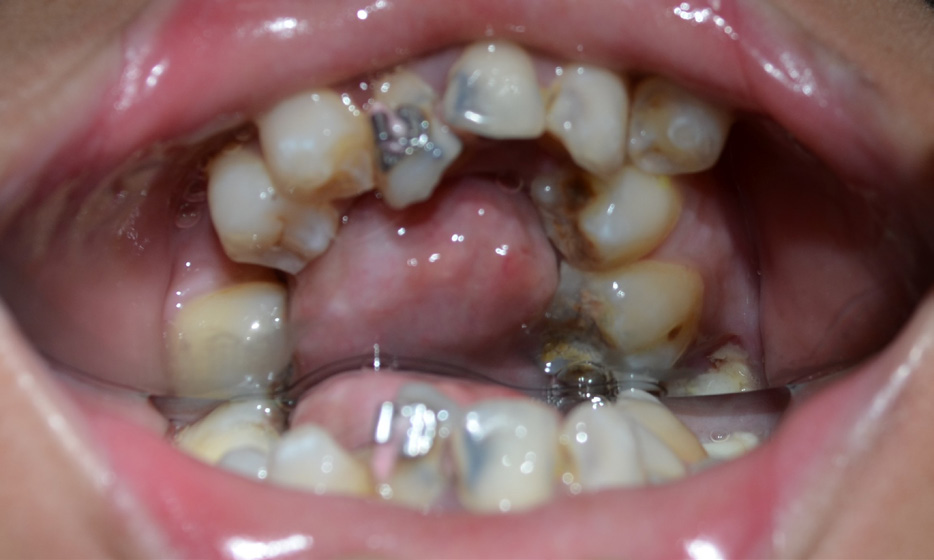
Intra oral picture showing prominent lingual bulge.

**Figure 3:**
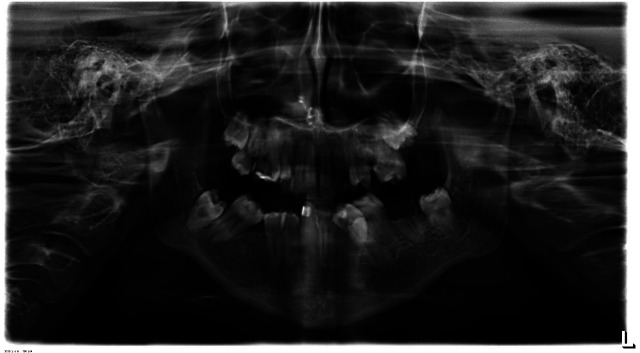
OPG.

A 22-year-old female patient reported to a clinic in Kolkata with the chief complain of irregularly arranged teeth. The patient was well coordinated, had normal growth and showed normal mental development for her age. No family history of any genetic and systemic disease or consanguineous marriage in the family was present. Functional examination did not reveal significant abnormality however mouth opening was reduced (32mm). She did not have complain of any respiratory compromise. Past dental history revealed her 14, 24 and carious 26 and 46 were extracted at the age of 12 years. Extra oral (Frontal) examination revealed a well-balanced proportionate and symmetrical face with incompetent lips. Normal intercanthal distance was observed and equal fifths of face was seen. Profile examination revealed equal thirds of face, normally positioned maxilla and retrognathic mandible with reduced neck chin length. Slightly reduced nasolabial angle suggestive of proclined upper incisors was observed. On intraoral examination a small rudimentary tongue (Fig. 1) with reduced range of movement was seen. Bulbous, raised and enlarged sublingual soft tissue was seen. Clinically missing 31,32 and 41 and retained roots of 37 was seen. Mild constriction was observed in the maxillary arch in the canine premolar region . Severely constricted mandibular arch with crowding of teeth and lingually tipped molars and upright incisors was observed (Fig. 2). Radiologic assessment OPG (Fig. 3) showed missing 14, 24, 26,31,32, 41 and 46 and retained roots of 37.

## Discussion

In 1971 Hall studied the association of hypoglossia with limb abnormalities and named it the oromandibular limb hypogenesis syndrome (7). The only essential criteria for this syndrome according to him was hypoglossia. He divided the syndrome into 5 broad categories in which hypoglossia without limb abnormality was put in Type 1A (4). The classification given by Hall in 1971 is the most widely accepted one till date ( Table 1) (2). Whether hypoglossia is syndromic or non-syndromic common features like micrognathia, severe transverse deficiency of mandible, hypodontia, crowding of teeth and functional adaptation like marked enlargement of the sublingual muscular ridge, hypertrophy of sublingual and submandibular glands are usually associated (3).

Tongue aids in various functions like suckling, mastication, swallowing, speech and has a role in development of the jaws (8). Cause of hypoglossia or aglossia is still not well understood (9). However, both genetic and environmental causes have been suggested by many authors. Genetic cause was suggested as few intrafamilial cases of hypoglossia have been reported. Environmental factors like teratogenic drugs, radiation, hyperthermia, intrauterine trauma have been suggested (10). No relevant history of any such exposure could be given by patient in this case. In spite of deficient tongue patient usually do not have speech and swallowing difficulties and these activities improve with age (7). In this case the patient did not have speech problem as could be elicited from her pronunciation also the patient reported at a late age and was well built and of normal physique suggesting she did not have swallowing difficulty. This is due to adaptation by hypertrophy of muscles and lingual mucosal tissues presenting as bulging in the floor of mouth. (Umeda *et al*.) reported articulation function and the swallowing function of a patient with hypoglossia-hypodactylia syndrome who was followed up to eight years old (10). The growth of jaws was compromised leading to micrognathia of the mandible which may be due to lack of mechanical stimulus from tongue. Most the cases of Oro Mandibular Limb Hypogenesis syndrome have reported presence of intra oral bands (4) however it was not present in this case. Mandibular molars were lingually tipped due to inadequate tongue size and thus inadequate lateral pressure exerted on the teeth.

Management of hypoglossia - No definitive treatment of hypoglossia can be suggested as the presentation is variable in different patients and so is the functional disability and in many patients functional adaptation is seen. Artificial tongue has been tried by few people but it was not successful. In this case patient had good functional adaptation and did not have any problem in mastication and swallowing due to the swelling in the floor of mouth. Taste perception according to the patient was also not a concern which is usually seen in most of the patients with hypoglossia. It is suggested that this happens because of presence of taste buds in mucosa of the floor of mouth (11). Her main concern was her facial appearance and dental crowding. After clinical assessment cephalometric evaluation and study model analysis three treatment options were presented to the patient in the order mentioned below

-Treatment options 

1) A combination of orthodontics and orthognathic surgery: a presurgical decompensation followed by mandibular advancement(surgical) and post-surgical finishing and settling

2) Orthodontics in conjunction with mandibular advancement using distraction osteogenesis 

3) Orthodontic camouflage: orthodontic alignment of the arches followed by distalisation of entire maxillary dentition to mask the present Class II situation due to mandibular deficiency

The patient however rejected the first two treatment options on account of being invasive and traumatic and wanted to go for less invasive modality so the third option was suggested and the limitations of this option was also explained. The patient opted for orthodontic camouflage. The patient is currently undergoing treatment with fixed orthodontic mechanotherapy using MBT 0.022 prescription.

## Conclusions

Such cases are very rare and thus the management and treatment of such cases become challenging for a practitioner. Presentation of such a case helps in adding to the knowledge bank and aid in exploring the various treatment options by guiding as a reference.

## Figures and Tables

**Table 1:** Halls classification of oro mandibular limb hypogenesis syndrome.

Type I	Hypoglossia Aglossia
Type II	Hypoglossia- hypodactylia Hypoglossia – hypomelia Hypoglossia - hypodactylomelia
Type III	Glossopalatine ankylosis With Hypoglossia With Hypoglossia- hypodactylia With Hypoglossia- hypomelia With Hypoglossia- hypodactylomelia
Type IV	Intraoral bands and fusion With Hypoglossia With Hypoglossia- hypodactylia With Hypoglossia- hypomelia With Hypoglossia- hypodactylomelia
Type V	Hanhart syndrome Charlie M syndrome Pierre- Robin syndrome Mobius syndrome Amniotic band syndrome

## Data Availability

The datasets used and/or analyzed during the current study are available from the corresponding author.
